# Influence of tumour size on the efficacy of targeted alpha therapy with ^213^Bi-[DOTA^0^,Tyr^3^]-octreotate

**DOI:** 10.1186/s13550-016-0162-2

**Published:** 2016-01-20

**Authors:** Ho Sze Chan, Mark W. Konijnenberg, Erik de Blois, Stuart Koelewijn, Richard P. Baum, Alfred Morgenstern, Frank Bruchertseifer, Wouter A. Breeman, Marion de Jong

**Affiliations:** Department of Nuclear Medicine, Erasmus Medical Center, Rotterdam, The Netherlands; Department of Radiology, Erasmus Medical Center, Rotterdam, The Netherlands; Institute for Transuranium Elements (ITU), Joint Research Centre, European Commission, Karlsruhe, Germany; Department of Nuclear Medicine/Center for PET/CT, Zentralklinik, Bad Berka, Germany

## Abstract

**Background:**

Targeted alpha therapy has been postulated to have great potential for the treatment of small clusters of tumour cells as well as small metastases. ^213^Bismuth, an α-emitter with a half-life of 46 min, has shown to be effective in preclinical as well as in clinical applications. In this study, we evaluated whether ^213^Bi-[DOTA^0^, Tyr^3^]-octreotate (^213^Bi-DOTATATE), a ^213^Bi-labelled somatostatin analogue with high affinity for somatostatin receptor subtype 2 (SSTR_2_), is suitable for the treatment of larger neuroendocrine tumours overexpressing SSTR_2_ in comparison to its effectiveness for smaller tumours. We performed a preclinical targeted radionuclide therapy study with ^213^Bi-DOTATATE in animals bearing tumours of different sizes (50 and 200 mm^3^) using two tumour models: H69 (human small cell lung carcinoma) and CA20948 (rat pancreatic tumour).

**Methods:**

Pharmacokinetics was determined for calculation of dosimetry in organs and tumours. H69- or CA20948-xenografted mice with tumour volumes of approximately 120 mm^3^ were euthanized at 10, 30, 60 and 120 min post injection of a single dose of ^213^Bi-DOTATATE (1.5–4.8 MBq). To investigate the therapeutic efficacy of ^213^Bi-DOTATATE, xenografted H69 and CA20948 tumour-bearing mice with tumour sizes of 50 and 200 mm^3^ were administered daily with a therapeutic dose of ^213^Bi-DOTATATE (0.3 nmol, 2–4 MBq) for three consecutive days. The animals were followed for 90 days after treatment. At day 90, mice were injected with 25 MBq ^99m^Tc-DMSA and imaged by SPECT/CT to investigate possible renal dysfunction due to ^213^Bi-DOTATATE treatment.

**Results:**

Higher tumour uptakes were found in CA20948 tumour-bearing animals compared to those in H69 tumour-bearing mice with the highest tumour uptake of 19.6 ± 6.6 %IA/g in CA20948 tumour-bearing animals, while for H69 tumour-bearing mice, the highest tumour uptake was found to be 9.8 ± 2.4 %IA/g. Nevertheless, as the anti-tumour effect was more pronounced in H69 tumour-bearing mice, the survival rate was higher. Furthermore, in the small tumour groups, no regrowth of tumour was found in two H69 tumour-bearing mice and in one of the CA20948 tumour-bearing mice. No renal dysfunction was observed in ^213^Bi-DOTATATE-treated mice after the doses were applied.

**Conclusions:**

^213^Bi-DOTATATE demonstrated a great therapeutic effect in both small and larger tumour lesions. Higher probability for stable disease was found in animals with small tumours. ^213^Bi-DOTATATE was effective in different neuroendocrine (H69 and CA20948) tumour models with overexpression of SSTR_2_ in mice.

**Electronic supplementary material:**

The online version of this article (doi:10.1186/s13550-016-0162-2) contains supplementary material, which is available to authorized users.

## Background

Peptide receptor radionuclide therapy (PRRT) is an effective treatment option for patients with an inoperable neuroendocrine tumour (NET) or with metastatic disease [1, 2]. Approximately 80 % of patients with NET show great anti-tumour response upon PRRT. The most commonly used radiolabelled peptides for treatment in NET are ^177^Lu-[DOTA^0^, Tyr^3^]- octreotate (^177^Lu-DOTATATE) and ^90^Y-[DOTA^0^, Tyr^3^]- octreotide (^90^Y-DOTATOC). ^90^Y and ^177^Lu are β-particles emitting radionuclides, with a maximum tissue penetration of 12 and 2 mm, respectively. For treatment of small tumour clusters and metastases, β-emitters may lack efficacy due to the relatively large tissue penetration and low linear energy transfer (LET). Targeted alpha therapy (TAT) with α-emitters has been described to be more suitable for these purposes, since the path length in tissue of α-particles is relatively short (in tissue 50–100 μm) compared to that of β-particles. Moreover, α-particles have a much higher LET (~100 keV/μm) and are therefore more powerful than β-particles (0.2 keV/μm) for therapeutic applications [3]. Bismuth-213 (^213^Bi), an α-emitter with a *T*_1/2_ of 45.6 min, has already been proven to be effective in TAT [4–7]. Recently, a clinical trial showed a promising anti-tumour effect in patients with progressive advanced neuroendocrine liver metastasis refractory to ^90^Y-DOTATOC or ^177^Lu-DOTATOC treatment after treatment with ^213^Bi-DOTATOC [8]. TAT with ^213^Bi-DOTATOC was able to overcome the resistance against β radiation in patients with different neuroendocrine tumours, in metastasis and in primary tumours, resulting in a high number of long-lasting anti-tumour responses.

The objective of this study was to investigate the therapeutic efficacy of ^213^Bi-DOTATATE in tumours of different sizes in mice (small vs large). Two different tumour models with somatostatin receptor subtype 2 (SSTR_2_) expression were used: CA20948 (rat pancreatic tumour) and H69 (human small cell lung carcinoma). Both tumours have relatively high SSTR_2_ expression, and both have often been applied as a model in preclinical studies of PRRT in NET [9–11]. The radiation sensitivity of CA20948 for low-LET radiation has been determined yielding *α*/*β* = 8.3 Gy [12]. The H69 cell line shows a higher radiation sensitivity *α*/*β* = 2.3 Gy [13]. Radiation damage by the high-LET α radiation from ^213^Bi is expected to follow a linear (-exponential) curve which was comparable for both cell lines.

For PRRT, the dose-limiting organs are bone marrow and kidneys. ^213^Bi-DOTATATE shows accumulation in the kidneys, resulting in high radiation doses to the kidneys; in this study, we therefore determined the safe dose of radioactivity administered for TAT in this mouse model. Renal function was evaluated after TAT by SPECT/CT at different time points post treatment using ^99m^Tc-DMSA (dimercaptosuccinic acid), a renal marker for tubular damage.

## Methods

### ^213^Bi-eluate

A ^213^Bi/^225^Ac generator (222 MBq) was provided by the Institute for Transuranium Elements (Karlsruhe, Germany). Prior to elution, the generator was rinsed using 0.01 M HCl (3 mL) and eluted using 0.1 M/0.1 M HCl/NaI (0.6 mL). After elution, the generator was rinsed with 0.01 M HCl and stored in 0.01 M HCl [4]. The activity of ^213^Bi was determined using a germanium detector (MetorX, Genie 2000 software, Canberra) [14].

### ^213^Bi-DOTATATE labelling

Eluate containing ^213^Bi (0.1 M/0.1 M in HCl/NaI) was added to a solution containing 7.0 nmol DOTATATE, 0.15 M TRIS buffer and 2.6 mM ascorbic acid in a total reaction volume of 800 μL. The reaction mixture was incubated for 5 min at 95 °C followed by 5 min at room temperature. To chelate any unbound/free ^213^Bi, 50 nmol DTPA (Sigma Aldrich, Zwijndrecht, The Netherlands) was added (manuscript submitted). Analysis by instant thin-layer chromatography (ITLC, Varian) and reverse-phase high-performance liquid chromatography (HPLC, 2695 separation module, Alliance, Waters, Etten-Leur, The Netherlands) of the labelled peptide was performed to determine the incorporation yield (%) and radiochemical purity (RCP) expressed as percentage of labelled peptide of interest compared to other detectable compounds [15]. The whole labelling procedure took on average 20 min to produce a ready-to-inject vial.

### ^99m^Tc-DMSA labelling

The ^99m^Tc-DMSA kit was purchased from Mallinckrodt (Petten, the Netherlands) and labelled according to the indicated procedure.

### Cell culture

The rat pancreatic tumour cell line CA20948 (derived from a rat pancreas tumour at our institute) with high SSTR_2_ expression was cultured in DMEM (Gibco, Life Technologies) supplemented with 10 % heat-inactivated fetal bovine serum. H69 tumour cells, human small cell lung carcinoma cells with SSTR_2_ expression (American Tissue Culture Collection, Wesel, Germany), were cultured in RPMI 1640 (Gibco, Life Technologies) supplemented with 10 % heat-inactivated fetal bovine serum and 50 IU/mL penicillin/streptomycin. All cells were cultured at 37 °C in a 5 % CO_2_/humidified air atmosphere.

### Animal models

All animal studies were in agreement with the Animal Welfare Committee requirements of Erasmus MC and conducted in accordance with accepted guidelines. Male 6–8-week-old BALB/c (nu/nu) nude mice were obtained from Charles River (Kißlegg, Germany). Subcutaneous tumours were established in mice by inoculation of approximately 5 × 10^6^ cells (CA20948 or H69), suspended in 200 μL sterile culture medium Hanks’ balanced salt solution (HBSS) in the case of CA20948 cells. Three days after inoculation of CA20948 cells, a tumour with a tumour size ~50 mm^3^ (used for the “smaller” tumour-size group) was reached, and 2 weeks after inoculation, a tumour size ~200 mm^3^ (used for the “larger” tumour-size group) was reached. For H69 cells, 200 μL, containing 1/3 Matrigel and 2/3 HBSS medium (Gibco, Life Technologies), was used. The cells were injected subcutaneously (s.c.) into the right flank of the mice. One week after inoculation of tumour cells, tumours with a volume of ~50 mm^3^ (used for the smaller tumour-size group) could be detected, and after 3 weeks, tumour volumes reached ~200 mm^3^ (used for the larger tumour-size group). Tumour volumes were estimated using a calliper.

### Pharmacokinetics of ^213^Bi-DOTATATE as function of time in different tumour models

When tumour volumes reached ~120 mm^3^, animals were divided into five groups with seven animals in each group for both H69 and CA20948 tumour-bearing animals. Four groups of mice received a single intravenous (i.v.) injection of ^213^Bi-DOTATATE (200 μL) via the tail vein, 3.8 ± 0.6 MBq/0.3 nmol/200 μL for H69 tumour-bearing animals and 2.1 ± 0.2 MBq/0.3 nmol/200 μL for CA20948 tumour-bearing animals. At 10, 30, 60 and 120 min post injection (p.i.), animals in the four groups were euthanized and tissues and organs of interest (including tumour, kidney, pancreas, spleen, liver, muscle, blood, femur, femur marrow and adrenals) were collected and counted using a gamma counter (Wallac Wizard 3, PerkinElmer, USA). Control animals received a single injection of ^213^Bi-DOTATATE plus an excess of DOTATATE (10^−6^ M) i.v. via the tail vein (blocking dose) to determine the non-receptor-specific binding of ^213^Bi-DOTATATE. The animals of the control group were euthanized 60 min p.i.; tissues and organs of interest were harvested and counted using a gamma counter. Data was expressed in mean percentage of total injected radioactivity per gram tissue (%IA/g) ± SD.

### Targeted alpha therapy

Tumour cells were inoculated into the right flank of the mice, and the tumours were allowed to grow until tumour volumes of approximately 50 and 200 mm^3^ were reached, respectively. On the day of treatment (*T* = 0 days), the animals (H69-bearing animals: small tumour *n* = 10, large tumour *n* = 7; CA20948-bearing animals: small tumour *n* = 8, large tumour *n* = 5) were injected i.v. via the tail vein with ^213^Bi-DOTATATE (2–4 MBq/0.3 nmol/200 μL) for three consecutive days. The control group (H69-bearing animals: small tumour *n* = 10, large tumour *n* = 9; CA20948-bearing animals: small tumour *n* = 5, large tumour *n* = 5) received i.v. via the tail vein three times 0.3 nmol/200 μL of unlabelled DOTATATE for three consecutive days.

All animals were followed for 90 days after treatment. The behaviour and the health status of the animals were observed daily, and the weight of the animals and the tumour volumes were monitored twice a week.

### Determination of renal function by ^99m^Tc-DMSA imaging

Renal function imaging was performed by a four-headed multi-pinhole SPECT/CT camera (Bioscan Inc., Washington D.C., USA). For ^99m^Tc imaging, an energy peak at 140 keV was selected with an energy window width ± 10 %. A nine-pinhole aperture (diameter of each pinhole 1.4 mm) was used on each camera head. For the CT topogram, an axial length of 27 mm was used to cover the renal region. Animals were imaged under anaesthesia by isoflurane and O_2_. The body temperature of the animals was maintained using a heated bed. Renal imaging of H69 (control *n* = 4, ^213^Bi-DOTATATE treated *n* = 8) and CA20948 tumour-bearing animals (control *n* = 2, ^213^Bi-DOTATATE treated *n* = 4) was performed 2 h after i.p. administration of ^99m^Tc-DMSA (25 MBq). ^99m^Tc-DMSA images were acquired for 12 min(20 projections, 60 s/projection) [16].

### Absorbed dose in organs

The absorbed radiation dose (defined as the mean absorbed dose in Gy) in organs and tumours was estimated from the results obtained in the biodistribution studies as described by Konijnenberg et al. [17] in a spherical geometry using Monte Carlo code MCNP5 (version 1.4) for the electrons and γ-rays [18] and MCNPX (version 2.5) [19] for the α-particles, all energy emission spectra were taken from ICRP database [20], and particle histories of 1 × 10^7^ was used for simulation to reduce the variation (<5 %) in the data. The mean absorbed energies for the 10 MBq ^213^Bi-DOTATATE injection were calculated for spheres of 1, 10, 100, 200, 300 and 500 mg containing tissue with a mass density of 1 g/mL to determine the spherical node *S*-values. A homogeneous activity distribution over the sphere was assumed. The absorbed dose *D*_*i*_ was calculated for each volume *i* according to the MIRD schema:

$$ {D}_i=\overset{\sim }{A_i}\times S\left(i\leftarrow i\right) $$

with *Ã*_*i*_ the time-integrated activity (TIA) in organ *i* and *S*(*i ← i*) the absorbed dose rate per unit activity (*S*-value). Only the self-dose *S*-values were considered and were obtained by interpolation between the sphere *S*-values for the measured organs or tumour mass. For small tumours, a minimum tumour mass of 50 mg was used for the absorbed dose calculation. The *Ã*_*i*_ in each organ was determined for ^213^Bi and its daughters taking the biodistribution into account according to the methods shown in Additional file 1. The absorbed dose to the kidneys was calculated both with the spherical *S*-values and with the nephron model by Hobbs et al. [21]. Three hypothetical activity distributions were considered for this heterogeneous model: (1) homogeneous in the cortex, (2) proximal tubular cell uptake and (3) glomerular cell uptake.

### Statistics

Statistical analysis was performed using *t* test and using the nonparametric Mann-Whitney test. *P* values <0.05 were considered statistically significant. For statistical significances in tumour growth patterns, one-way ANOVA was used. Values are presented as the mean and the standard deviation (SD).

## Results

An incorporation yield of ≥99 % and a RCP ≥ 85 % were found for the radiopeptide, as determined by ITLC and HPLC, respectively.

### Pharmacokinetic studies

**Fig. 1:**
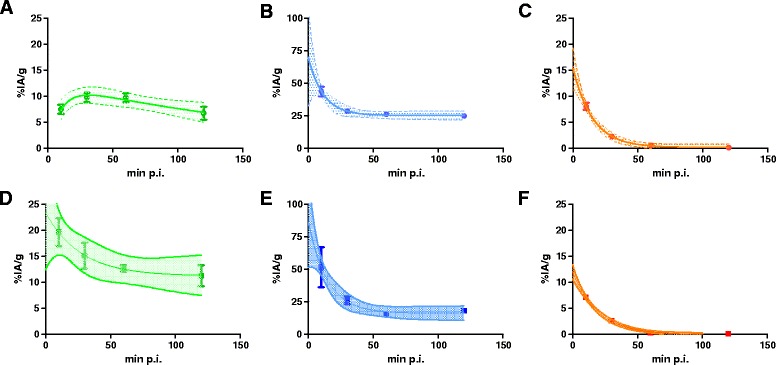
**a**–**f** Selected pharmacokinetics of ^213^Bi-DOTATATE from Tables 1 and 2. ^213^Bi-DOTATATE pharmacokinetics in H69 tumour-bearing mice, uptake in tumours (**a**) and kidney (**b**), and radioactivity in blood (**c**) are shown over time in H69 tumour-bearing animals. Same data are shown for CA20948 tumour-bearing animals: uptake in tumours (**d**) and kidneys (**e**) and radioactivity in blood (**f**). *Solid curves* show the mean uptake value expressed in %IA/g ± SD. The *dotted curves* indicate the 95 % confidence interval

Biodistribution studies using ^213^Bi-DOTATATE in H69 tumour-bearing mice revealed relatively high tumour uptake. The highest tumour uptake was reached at 30 min p.i., 9.8 ± 2.4 %IA/g. Tumour uptake *T*_1/2_ was 7 ± 2 min, and tumour clearance *T*_1/2_ was 2.3 ± 1.1 h. An even higher renal activity compared to tumour uptake was found at all time points, the highest renal activity being found at 10 min p.i., 44 ± 10 %IA/g. At 120 min p.i., the renal activity still was 25 ± 3 %IA/g. Blood clearance in H69 tumour-bearing animals was fast, and clearance *T*_1/2_ was 11 ± 2 min, see Fig. 1a–c. All other organs had relative low uptake at 120 min p.i., < 2 %IA/g, see Table 1.

**Table 1:** Pharmacokinetics of ^213^Bi-DOTATATE at 10, 30, 60 and 120 min post injection (p.i.) in H69 tumour-bearing animals, expressed in mean %IA/g ± SD, *n* = 7

Uptake (%IA/g)	10 min p.i.	30 min p.i.	60 min p.i.	120 min p.i.
Tumour	7.5 ± 2.2	9.8 ± 2.4	9.8 ± 2.3	6.8 ± 3.2
Muscle	2.1 ± 0.8	1.1 ± 0.2	1.0 ± 1.4	0.5 ± 0.4
Kidney	44.0 ± 9.6	28.5 ± 3.7	26.2 ± 2.6	25.0 ± 3.8
Pancreas	3.2 ± 0.3	2.8 ± 0.3	1.8 ± 0.2	1.4 ± 0.2
Liver	2.1 ± 0.8	0.9 ± 0.09	0.4 ± 0.06	0.3 ± 0.05
Blood	8.1 ± 1.8	2.3 ± 1.1	0.6 ± 0.2	0.1 ± 0.06
Spleen	2.3 ± 0.6	1.0 ± 0.09	0.4 ± 0.07	0.2 ± 0.07
Adrenal	3.6 ± 1.2	2.0 ± 0.6	0.9 ± 0.4	0.7 ± 0.4
Femur	2.8 ± 0.4	1.5 ± 0.4	1.0 ± 0.5	0.4 ± 0.4

%IA/g = mean percentage of total injected radioactivity per gram tissue

Tumour uptake of ^213^Bi-DOTATATE in CA20948 tumour-bearing mice was already highest at 10 min p.i., 19.6 ± 6.6 %IA/g, and the tumour uptake *T*_1/2_ was therefore unable to estimate. A clearance *T*_1/2_ of 2.2± 0.9 h was found for CA20948 tumours. Similar renal activity was found in CA20948 tumour-bearing animals. Renal activity was highest at 10 min p.i., 52 ± 17 %IA/g. Renal clearance *T*_1/2_ was 10 ± 3 min, whereas blood clearance *T*_1/2_ was 14 ± 1 min, see Fig. 1d–f. At 120 min p.i., uptakes in other organs remained low, < 2 %IA/g, see Table 2.

**Table 2:** Pharmacokinetics of ^213^Bi-DOTATATE at 10, 30, 60 and 120 min post injection (p.i.) in CA20948 tumour-bearing animals, expressed in mean %IA/g ± SD, *n* = 7

Uptake (%IA/g)	10 min p.i.	30 min p.i.	60 min p.i.	120 min p.i.
Tumour	19.6 ± 6.6	15.1 ± 6.6	12.7 ± 1.6	11.2 ± 5.4
Muscle	3.1 ± 0.8	1.5 ± 0.8	1.4 ± 1.9	0.2 ± 0.2
Kidney	51.5 ± 16.6	26.0 ± 3.3	15.0 ± 1.5	18.0 ± 1.9
Pancreas	3.6 ± 0.2	3.1 ± 0.4	1.5 ± 0.3	1.2 ± 0.2
Liver	2.0 ± 0.2	1.0 ± 0.1	0.2 ± 0.04	0.2 ± 0.03
Blood	7.1 ± 1.0	2.6 ± 0.4	0.3 ± 0.2	0.07 ± 0.04
Spleen	2.0 ± 0.2	1.1 ± 0.4	0.2 ± 0.05	0.2 ± 0.04
Adrenal	3.4 ± 0.7	2.2 ± 0.8	0.6 ± 0.1	0.4 ± 0.1
Femur	2.4 ± 0.2	1.4 ± 0.2	0.6 ± 0.1	0.2 ± 0.04

%IA/g = mean percentage of total injected radioactivity per gram tissue

### Dosimetry

Dosimetry calculations in both tumour models were based on the data obtained from the biodistribution studies at 10, 30, 60 and 120 min p.i. In H69 tumour-bearing mice, a relatively high tumour-absorbed radiation dose was estimated: 0.45 Gy/MBq injected ^213^Bi-DOTATATE calculated for 100 mg tumour. The renal-absorbed dose was four times as high as the tumour-absorbed radiation dose: 2 Gy/MBq injected ^213^Bi-DOTATATE. The absorbed radiation dose of ^213^Bi-DOTATATE in the pancreas, spleen and liver was low compared to those in the tumour and kidney, 0.15, 0.06 and 0.06 Gy/MBq, respectively. Similar results were observed in CA20948 tumour-bearing animals. A lower tumour-absorbed radiation dose was found than that in the kidneys: 0.78 Gy/MBq in the tumour vs 1.6 Gy/MBq. Absorbed radiation doses in the pancreas, spleen and liver were 0.13, 0.05 and 0.06 Gy/MBq, respectively. For both tumour-bearing animal models, an absorbed dose of < 0.02 Gy/MBq was found in the bone marrow.

**Fig. 2:**
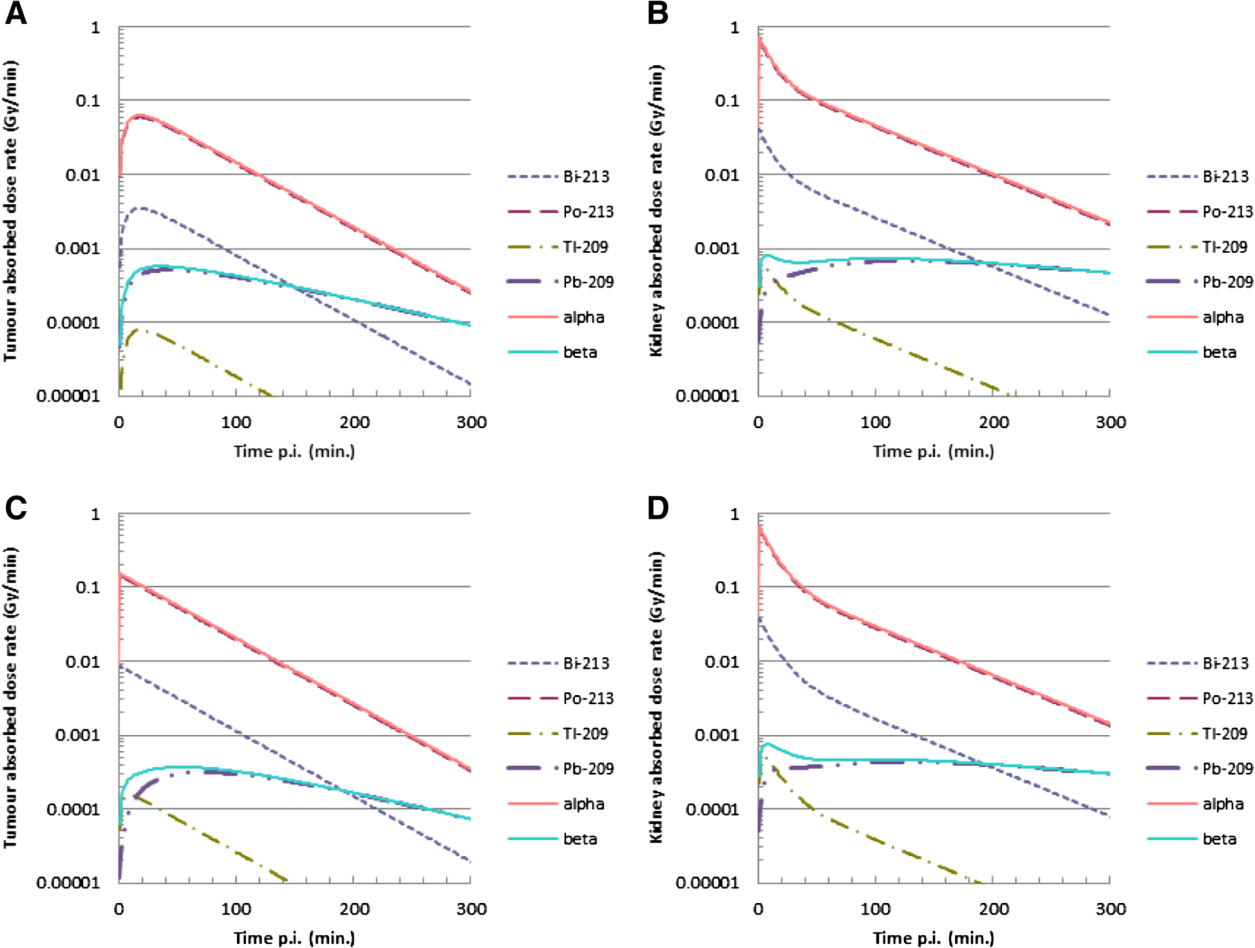
Calculated absorbed radiation dose rates in the tumour and kidney after 10 MBq ^213^Bi-DOTATATE, based on the data obtained in the biodistribution study. Absorbed dose rate as function of time in H69 tumour-bearing animals; tumour (**a**) and kidney (**b**). Same data are shown of the tumour (**c**) and kidney (**d**) for CA20948 tumour-bearing animals. *Red lines* indicate the absorbed dose rate contributed by all alpha particles and *blue lines* by all beta particles. The *dotted lines* indicate the individual absorbed dose rate of ^213^Bi, ^213^ Po, ^209^Tl and ^209^ Pb

Tumour- and kidney-absorbed radiation doses in H69 or CA20948 tumour-bearing animals were mainly caused by the α-particles emitted. The absorbed dose rate was higher in CA20948 than in H69 tumours, and the kidney-absorbed radiation dose was similar; see Fig. 2a–d. For an instantaneous uptake in the H69 tumour, its absorbed dose would rise by 12 % to 0.50 Gy/MBq, whereas the absorbed dose to the CA20948 tumour is less dependent on the uptake kinetics: the dose is lowered by 2 % to 0.77 Gy/MBq when the same uptake kinetics as for the H69 tumour is assumed.

### Targeted alpha therapy

**Fig. 3:**
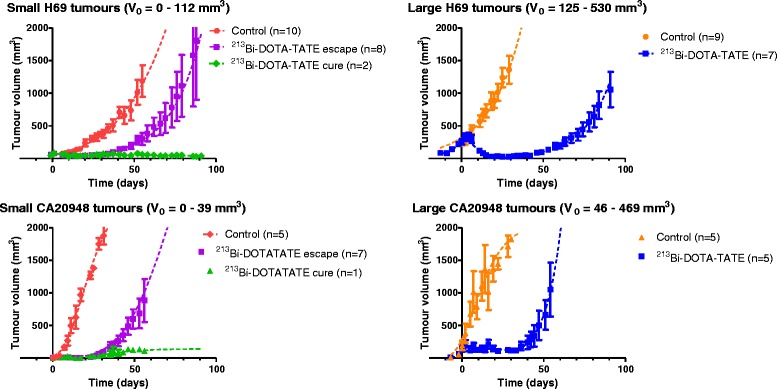
Overview of mean tumour volumes ± standard deviation (SD) in mm^3^ per group after treatment of ^213^Bi-DOTATATE (2–4 MBq per injection) or unlabelled DOTATATE (control) in H69 and CA20948 tumour-bearing mice for three consecutive days. *V*_0_ is the starting tumour volume. The *dotted curves* show the extrapolated growth curves of the tumours assuming 100 % survival of animals

Therapeutic anti-tumour effects were observed in all animals treated with ^213^Bi-DOTATATE, see Fig. 3. The tumour volume doubling time was 16 ± 6 days for the H69 and 10 ± 5 days for the CA20948 tumours, without any difference between the growth rate of small tumours and large tumours. The mean times to reach the end volume of 2000 mm^3^ of the control and treated animals of both tumour models are indicated in Table 3. For small tumours, tumour sizes < 50 mm^3^ could not be measured accurately; therefore, 50 mm^3^ was used as the minimum tumour sizes in our calculation and this was in three cases of H69 tumour-bearing animals with a small tumour and in all cases of CA20948 tumour-bearing animals with a small tumour. Overall calculated cumulative tumour-absorbed dose in small tumours was higher than large tumours. In CA20948 tumours, the cumulative tumour-absorbed dose was higher than in H69 tumours.

**Table 3:** An overview of mean time (days = d) to reach a tumour volume of 2000 mm^3^ after ^213^Bi-DOTATATE therapy

	H69 small	H69 large	CA20948 small	CA20948 large
Control	73 ± 19 d	38 ± 11 d	43 ± 20 d	27 ± 14 d
Therapy	102 ± 19 d	110 ± 19 d	76 ± 15 d	77 ± 25 d
Tumour dose (Gy)	5 ± 1 Gy	2.0 ± 0.6 Gy	17 ± 1 Gy	10 ± 5 Gy
Growth delay (control vs therapy)	28 ± 9 d***	73 ± 24 d*	33 ± 17 d NS	50 ± 13 d**

*NS* not significant

* = *P* value ≤0.05; ** = *P* value ≤0.01; *** = *P* value ≤0.001

The tumour dose (Gy) ± SD was estimated based on the tumour size at *T* = 0 of therapy and the injected activity (MBq). Growth delay (days) was the Δ*T* for tumour to reach 2000 mm^3^ of therapy group vs control group

Shrinkage of tumours was observed in H69 tumour-bearing animals with large tumours and a delay of tumour growth around 70 days after ^213^Bi-DOTATATE (cumulative injected activity of 9.4 ± 0.7 MBq) treatment. In CA20948 tumour-bearing animals with large tumours, the inhibition of tumour growth was initiated around 30 days after therapy (cumulative injected activity 10.2 ± 0.4 MBq ^213^Bi-DOTATATE), leading to a tumour growth delay time of 50 days. Similar results were found in CA20948 tumour-bearing animals with small tumours after therapy (cumulative injected activity 7.7 ± 0.4 MBq); a delay in tumour growth of around 30 days was observed, and in one of the animals, no tumour regrowth was observed. The delay time was estimated excluding the animals with cure or stable disease. In H69 tumour-bearing animals with small tumours, a delay of tumour growth was found for around 30 days after therapy (cumulative injected activity 7.4 ± 0.5 MBq), and in two animals, no tumour regrowth was observed until the end of the study at day 90. Here, the two animals with cure/stable disease were excluded from the estimated delay time.

### Survival

**Fig. 4:**
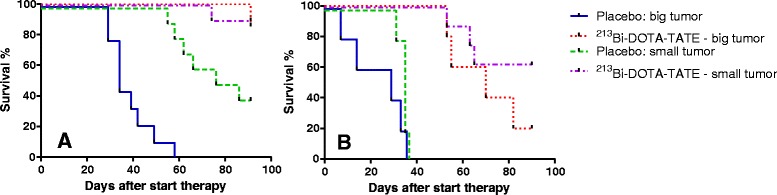
Survival (percentage) after ^213^Bi-DOTATATE or DOTATATE treatment in **a** H69 tumour-bearing and **b** CA20948 tumour-bearing mice

All animals treated with ^213^Bi-DOTATATE showed a higher survival rate than the control animals, see Fig. 4. In the control, non-TAT H69 tumour-bearing animals, a median survival of 34 days was found for animals bearing large tumours and 81 days for animals bearing small tumours. H69 tumour-bearing animals with small tumours had a median survival of > 90 days after ^213^Bi-DOTATATE therapy (control vs treated, *P* value = <0.0001). Similar results were obtained in H69 tumour-bearing animals with large tumours after TAT; a median survival > 90 days was found (control vs treated, *P* value = 0.0195). In the CA20948 tumour-bearing animals without TAT, a median survival of 29 days was found for animals bearing large tumours, whereas for animals bearing small tumours, this was 35 days. After ^213^Bi-DOTATATE, a median survival of 70 days was found in the large-tumour-animal group (control vs treated, *P* value = not significant) and > 90 days in the small-tumour-animal group (control vs treated, *P* value = 0.005).

### ^99m^Tc-DMSA uptake in the kidney

**Fig. 5:**
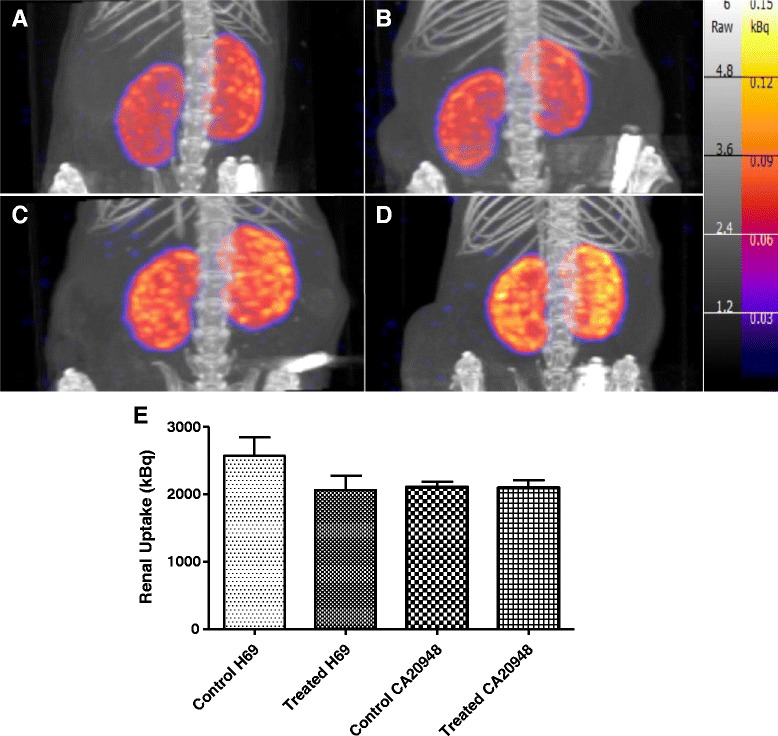
**a**–**d** ^99m^Tc-DMSA renal SPECT images in H69 control (**a**), ^213^Bi-DOTATATE-treated H69 mice (**b**), CA20948 control (**c**) and ^213^Bi-DOTATATE-treated CA20948 mice (**d**). **e** Renal uptake (in kBq) of ^99m^Tc-DMSA (25 MBq) in H69 and CA20948 tumour-bearing mice after therapy with ^213^Bi-DOTATATE or DOTATATE

Renal function was investigated in H69 and CA20948 tumour-bearing animals at day 90 after ^213^Bi-DOTATATE therapy or unlabelled DOTATATE treatment. Renal function was determined by renal uptake of ^99m^Tc-DMSA (25 MBq). No significant differences in ^99m^Tc-DMSA uptake in the kidneys were observed in ^213^Bi-DOTATATE-treated mice and control animals for both tumour models. In H69 tumour-bearing mice, a mean renal uptake of 2.6 ± 0.8 MBq (mean concentration of 2.9 ± 0.6 kBq/mm^3^) in the control group (*n* = 4) vs 2.1 ± 0.9 MBq (mean concentration of 2.9 ± 0.8 kBq/mm^3^) in ^213^Bi-DOTATATE-treated animals (*n* = 8) was found. Similar results were obtained in CA20948 tumour-bearing animals: mean uptake of 2.1 ± 0.2 MBq (mean concentration of 3.1 ± 0.4 kBq/mm^3^) in ^213^Bi-DOTATATE-treated animals (*n* = 4) and 2.1 ± 0.3 MBq (mean concentration 3.2 ± 0.3 kBq/mm^3^) in control (*n* = 2) were found. Figure 5a–e shows ^99m^Tc-DMSA uptake in kidneys of both tumour models.

## Discussion

In this study, we compared the efficacy of ^213^Bi-DOTATATE TAT in tumours of different sizes in two different tumour models, both with expression of SSTR_2_. ^213^Bi-DOTATATE showed a very good therapeutic effect in both small and large tumours in the two different tumour models. Tumour-absorbed doses (Gy) in CA20948 tumour-bearing animals were higher compared to those in H69 tumour-bearing mice. Despite these findings, ^213^Bi-DOTATATE showed greater therapeutic effects in H69 tumour-bearing animals than in CA20948 tumour-bearing animals; a higher survival rate was observed in H69 tumour-bearing animals at the end of the study. However, no significant difference in tumour (re)growth inhibition time between the two tumour models was found, even though the absorbed dose in the tumour was higher in CA20948 tumour-bearing animals. In vitro, CA20948 cells appeared also more radioresistant than H69 cells; at external beam irradiation with 2 Gy, a survival fraction of H69 was 37 % [13] and for CA20498, 52 % was found [12].

In this study, we observed a higher anti-tumour effect in small tumours. In H69 tumour-bearing mice with small tumours, two animals showed stable disease and in eight animals, a partial response was found after treatment. In the case of CA20948 tumour-bearing animals with small tumours, one animal showed stable disease till the end of the study and partial response was found in eight of the animals after ^213^Bi-DOTATATE therapy. In H69 and CA20948 tumour-bearing animals with large tumours, a partial response was found. A longer tumour growth delay was found in animals with large tumours of both tumour models compared to small tumours. The time a tumour takes to reach 2000 mm^3^ in small tumours is longer than in large tumours; therefore, Δ delay time for tumour growth was used. Nevertheless, in animals showing regrowth, the small tumours were less responsive than the large tumours in both models.

The reason for partial remission can be explained as the distribution of the radiation-absorbed dose depends on the tumour size, the physical properties of the radionuclide and its possible inhomogeneous intratumoural distribution. In those cases where the tumour dimensions are smaller than the range of the ionizing particles, a large proportion of the absorbed energy can “escape”, resulting in a suboptimal effect of ^213^Bi-DOTATATE TAT [22]. In larger tumours, also pronounced therapeutic effects were observed after ^213^Bi-DOTATATE therapies in both tumour models. However, no complete remissions were observed in these animals. Besides tumour perfusion [23] and inhomogeneous distribution of SSTR_2_, tumour size [17, 22], vessel density, permeability and tumour growth doubling time can play a critical role for the success of PRRT; this is especially true for α-emitters, where the penetration depth in tissue is relatively short (50–100 μm). In large tumours, often heterogeneity of receptor distribution [24] is observed, resulting in insufficient efficacy of the treatment and leading to probability survival of tumour cells. Therefore multiple fractions of therapy might improve the effect in larger tumours [25], especially in the case of TAT.

For somatostatin receptor-targeted PRRT, nephrotoxicity is often the dose-limiting factor, since reabsorption of radiolabelled somatostatin analogues by cells in the proximal tubule of the nephron occurs [26].

In this study, the calculated renal-absorbed dose using our simplified model was compared to the model described by Hobbs et al. [21]. We assumed all renal uptakes to be localized in the proximal tubular cells and took the glomerular cells as target. The absorbed dose in the glomeruli was 57 % lower than for the mean absorbed kidney dose. On the other hand, the dose to the glomeruli is almost a factor 20 higher when all activity was concentrated in the glomerular cells. The homogeneous mean absorbed dose we calculated with the sphere model was 24 % higher than when calculated with the cortex model from Hobbs et al. [21].

^99m^Tc-DSMA can provide information on renal function after renal tubular damage and has been used as renal marker in PRRT [27]. In this study, we did not find any changes in renal function in animals after TAT (highest cumulative activity used was 11.5 MBq ^213^Bi-DOTATATE). Kidney histology was performed in CA20948-bearing animals (data not shown), and no significant differences were observed in control vs treated animals. However, ^213^Bi-DOTATATE is known to accumulate in the kidney, leading to a high renal-absorbed dose. In patients, the maximum tolerated dose in kidney is postulated to be 23–28 Gy [28–30]. Kidney protectants during TAT are therefore advised [9, 31, 32], especially when higher treatment activities are used. In another study, we found a maximum tolerated activity of 21.2 ± 1.7 MBq in the presence of i.p. l-lysine as kidney protectant prior to the ^213^Bi-DOTATATE treatment; in the absence of l-lysine, the maximum tolerated activity was <13.1 ± 1.2 MBq (manuscript in preparation).

Even though we have not been able to achieve complete remission of all tumours, this study demonstrated that TAT can be effective for the treatment of both large and small tumours. These findings are in line with those of Kratochwil et al. [8] who demonstrated the effectiveness of TAT in eight patients refractory to PRRT with ^177^Lu and ^90^Y. We therefore believe that TAT is most promising for the treatment of NET. In combination with PRRT using ^90^Y and ^177^Lu peptides [33, 34], even greater therapeutic efficacy might be achieved, especially for in the treatment of patients with larger NET.

## Conclusions

TAT with ^213^Bi-DOTATATE shows very good therapeutic efficacy for the treatment of both large and small tumours in these mouse models. In small tumours, ^213^Bi-DOTATATE demonstrated greater anti-tumour effect by its higher probability for inducing stable disease. Furthermore, TAT using ^213^Bi-DOTATATE demonstrated to be safe in our experimental setting, with kidneys as dose-limiting organs. ^213^Bi-DOTATATE therefore held great promise for targeted therapy for neuroendocrine tumours that express SSTR_2_.

## Ethics approval

All applicable international, national and/or institutional guidelines for the care and use of animals were followed.

## Additional file

Additional file 1:
**Biodistribution of** ^**213**^ **Bi activity and its daughters for single compartment kinetics.**
