# Supplementary material for: Influence of tumour size on the efficacy of targeted alpha therapy with 213Bi-[DOTA0,Tyr3]-octreotate
Source: EJNMMI Res. 2016 Jan 20;6:6. doi: 10.1186/s13550-016-0162-2 (PMC4720616; doi:10.1186/s13550-016-0162-2)
Supplement: Additional file 1: — Biodistribution of 213 Bi activity and its daughters for single compartment kinetics. [file 13550_2016_162_MOESM1_ESM.docx]

**Additional file 1**

Influence of tumour size on the efficacy of targeted alpha therapy with ^213^Bi-[DOTA^0^,Tyr^3^]-octreotate

Ho Sze Chan^1^, Mark W. Konijnenberg^1^, Erik de Blois^1^, Stuart Koelewijn^1^, Richard P.Baum^4^, Alfred Morgenstern^3^, Frank Bruchertseifer^3^, Wouter A. Breeman^1^, Marion de Jong^1,2^

1. Department of Nuclear Medicine, Erasmus Medical Center, Rotterdam, The Netherlands
2. Department of Radiology, Erasmus Medical Center, Rotterdam, The Netherlands
3. European Commission, Joint Research Centre, Institute for Transuranium Elements (ITU), Karlsruhe, Germany
4. Zentralklinik, Department of Nuclear Medicine/Center for PET/CT, Bad Berka, Germany

Biodistribution of ^213^Bi activity and its daughters for single compartment kinetics

1. **Single compartment clearance model for ^213^Bi conjugates activity in organs or tumours.**

The ^213^Bi activity in the volume is cleared with a biologic clearance constant *λ_b_* (*= ln(2)/T_b_,* with *T_b_* the clearance half-life) and the kinetics follows an exponential decay function:

$$A_{Bi}\left( t \right)=A_{Bi}(0)e^{-\left( \lambda_{Bi}+\lambda_{b} \right)t}$$

The activity integration from time 0 to T of the ^213^Bi activity and time integrated activity Ã_Bi_ is then:

$Ã_{Bi}\left( T \right)=\int_{0}^{T} A_{Bi}\left( t \right) dt= \frac{A_{Bi}(0)}{\lambda_{Bi}+\lambda_{b}}\left( 1-e^{-\left( \lambda_{Bi}+\lambda_{b} \right)T} \right)$ ⇒ $\lim_{T\to\infty} Ã_{Bi}(T)=Ã_{Bi}=\frac{A_{Bi}(0)}{\lambda_{Bi}+\lambda_{b}}$

^213^Bismuth decays with a branching ratio BR *(=0.9791)* to ^213^Po and the ^213^Po kinetics follows:

$$A_{Po}\left( t \right)={\frac{BR\lambda_{Po}}{\lambda_{Po}-\lambda_{Bi}}\times A}_{Bi}(0)\left( e^{-\left( \lambda_{Bi}+\lambda_{b} \right)t}-e^{-\left( \lambda_{Po}+\lambda_{b} \right)t} \right)$$

The activity integration over time of the ^213^Po activity and time integrated activity Ã_Po_ is then:

$Ã_{Po}\left( T \right)={\frac{BR\lambda_{Po}}{\lambda_{Po}-\lambda_{Bi}} A}_{Bi}(0)\left( \frac{1-e^{-\left( \lambda_{Bi}+\lambda_{b} \right)T}}{\lambda_{Bi}+\lambda_{b}}-\frac{1-e^{-\left( \lambda_{Po}+\lambda_{b} \right)T}}{\lambda_{Po}+\lambda_{b}} \right)$ ⇒ $Ã_{Po}={\frac{BR\lambda_{Po}}{\lambda_{Po}-\lambda_{Bi}} A}_{Bi}(0)\left( \frac{1}{\lambda_{Bi}+\lambda_{b}}-\frac{1}{\lambda_{Po}+\lambda_{b}} \right)$ ⇒$Ã_{Po}\approx BRA_{Bi}(0)\frac{1}{\lambda_{Bi}+\lambda_{b}}$

The decay and biodistribution of the other daughters ^209^Tl and ^209^Pb are described quite similarly:

$$A_{Tl}\left( t \right)={\frac{(1-BR{) \lambda}_{Tl}}{\lambda_{Tl}-\lambda_{Bi}}\times A}_{Bi}(0)\left( e^{-\left( \lambda_{Bi}+\lambda_{b} \right)t}-e^{-\left( \lambda_{Tl}+\lambda_{b} \right)t} \right)$$

with: $Ã_{Tl}\left( T \right)={\frac{(1-BR)\lambda_{Tl}}{\lambda_{Tl}-\lambda_{Bi}} A}_{Bi}(0)\left( \frac{1-e^{-\left( \lambda_{Bi}+\lambda_{b} \right)T}}{\lambda_{Bi}+\lambda_{b}}-\frac{1-e^{-\left( \lambda_{Tl}+\lambda_{b} \right)T}}{\lambda_{Tl}+\lambda_{b}} \right)$ ⇒

$Ã_{Tl}={\frac{(1-BR)\lambda_{Tl}}{\lambda_{Tl}-\lambda_{Bi}} A}_{Bi}(0)\left( \frac{1}{\lambda_{Bi}+\lambda_{b}}-\frac{1}{\lambda_{Tl}+\lambda_{b}} \right)$

$$A_{Pb}\left( t \right)={\frac{BR\lambda_{Po}\lambda_{Pb}}{\lambda_{Po}-\lambda_{Bi}}\times A}_{Bi}\left( 0 \right)\left( \frac{e^{-\left( \lambda_{Bi}+\lambda_{b} \right)t}-e^{-\left( \lambda_{Pb}+\lambda_{b} \right)t}}{\lambda_{Pb}-\lambda_{Bi}}-\frac{e^{-\left( \lambda_{Po}+\lambda_{b} \right)t}-e^{-\left( \lambda_{Pb}+\lambda_{b} \right)t}}{\lambda_{Pb}-\lambda_{Po}} \right)+$$

$$\frac{(1-BR)\lambda_{Tl}\lambda_{Pb}}{\lambda_{Tl}-\lambda_{Bi}}\times A_{Bi}(0)\left( \frac{e^{-\left( \lambda_{Bi}+\lambda_{b} \right)t}-e^{-\left( \lambda_{Pb}+\lambda_{b} \right)t}}{\lambda_{Pb}-\lambda_{Bi}}-\frac{e^{-\left( \lambda_{Tl}+\lambda_{b} \right)t}-e^{-\left( \lambda_{Pb}+\lambda_{b} \right)t}}{\lambda_{Pb}-\lambda_{Tl}} \right)$$

⇒

$$Ã_{Pb}\left( T \right)={\frac{BR\lambda_{Po}\lambda_{Pb}}{\lambda_{Po}-\lambda_{Bi}}\times A}_{Bi}\left( 0 \right)\left( \frac{\frac{\left. 1-e^{-\left( \lambda_{Bi}+\lambda_{b} \right)T} \right.}{\lambda_{Bi}+\lambda_{b}}-\frac{\left. 1-e^{-\left( \lambda_{Pb}+\lambda_{b} \right)T} \right.}{\lambda_{Pb}+\lambda_{b}}}{\lambda_{Pb}-\lambda_{Bi}}-\frac{\frac{{1-e}^{-\left( \lambda_{Po}+\lambda_{b} \right)T}}{\lambda_{Po}+\lambda_{b}}-\frac{{1-e}^{-\left( \lambda_{Pb}+\lambda_{b} \right)T}}{\lambda_{Pb}+\lambda_{b}}}{\lambda_{Pb}-\lambda_{Po}} \right)+$$

$$\frac{(1-BR)\lambda_{Tl}\lambda_{Pb}}{\lambda_{Tl}-\lambda_{Bi}}\times A_{Bi}(0)\left( \frac{\frac{\left. 1-e^{-\left( \lambda_{Bi}+\lambda_{b} \right)T} \right.}{\lambda_{Bi}+\lambda_{b}}-\frac{\left. 1-e^{-\left( \lambda_{Pb}+\lambda_{b} \right)T} \right.}{\lambda_{Pb}+\lambda_{b}}}{\lambda_{Pb}-\lambda_{Bi}}-\frac{\frac{{1-e}^{-\left( \lambda_{Tl}+\lambda_{b} \right)T}}{\lambda_{Tl}+\lambda_{b}}-\frac{{1-e}^{-\left( \lambda_{Pb}+\lambda_{b} \right)T}}{\lambda_{Pb}+\lambda_{b}}}{\lambda_{Pb}-\lambda_{Tl}} \right)$$

$$Ã_{Pb}={\frac{BR\lambda_{Po}\lambda_{Pb}}{\lambda_{Po}-\lambda_{Bi}}\times A}_{Bi}\left( 0 \right)\left( \frac{\frac{\left. 1 \right.}{\lambda_{Bi}+\lambda_{b}}-\frac{\left. 1 \right.}{\lambda_{Pb}+\lambda_{b}}}{\lambda_{Pb}-\lambda_{Bi}}-\frac{\frac{1}{\lambda_{Po}+\lambda_{b}}-\frac{1}{\lambda_{Pb}+\lambda_{b}}}{\lambda_{Pb}-\lambda_{Po}} \right)+$$

$$\frac{(1-BR)\lambda_{Tl}\lambda_{Pb}}{\lambda_{Tl}-\lambda_{Bi}}\times A_{Bi}(0)\left( \frac{\frac{\left. 1 \right.}{\lambda_{Bi}+\lambda_{b}}-\frac{\left. 1 \right.}{\lambda_{Pb}+\lambda_{b}}}{\lambda_{Pb}-\lambda_{Bi}}-\frac{\frac{1}{\lambda_{Tl}+\lambda_{b}}-\frac{1}{\lambda_{Pb}+\lambda_{b}}}{\lambda_{Pb}-\lambda_{Tl}} \right)$$

1. **Single compartment uptake and clearance model for ^213^Bi conjugates activity.**

Uptake kinetics in organ or tumour with rate constant *λ_u_* (*= ln(2)/T_u_,* with *T_u_* the uptake half-life) and clearance back to plasma with rate constant *λ_b_*

^213^Bi kinetics: $A_{Bi}\left( t \right)=\frac{\lambda_{u}}{\lambda_{u}-\lambda_{b}}A_{Bi}\left( 0 \right)e^{-\lambda_{Bi}t}(e^{-\lambda_{b}t}-e^{-\lambda_{u}t})$ ⇒

$$Ã_{Bi}\left( T \right)=\frac{\lambda_{u}}{\lambda_{u}-\lambda_{b}}A_{Bi}\left( 0 \right)(\frac{1-e^{-({\lambda_{Bi}+\lambda}_{b})T}}{{\lambda_{Bi}+\lambda}_{b}}-\frac{{1-e}^{-({\lambda_{Bi}+\lambda}_{u})T}}{{\lambda_{Bi}+\lambda}_{u}})$$

⇒ $Ã_{Bi}=\frac{A_{Bi}\left( 0 \right)\lambda_{u}}{\left( \lambda_{Bi}+\lambda_{b} \right){(\lambda}_{Bi}+\lambda_{u})}$

^213^Po kinetics:

$A_{Po}\left( t \right)=\frac{\lambda_{Po}\lambda_{u}BR}{{(\lambda}_{u}-\lambda_{b})(\lambda_{Po}-\lambda_{Bi})}A_{Bi}\left( 0 \right)\left( e^{-(\lambda_{Bi}+\lambda_{b})t}{-e}^{-(\lambda_{Bi}+\lambda_{u})t}-e^{-(\lambda_{Po}+\lambda_{b})t}+e^{-(\lambda_{Po}+\lambda_{u})t} \right)$ ⇒

$Ã_{Po}(T)=\frac{\lambda_{Po}\lambda_{u}BR}{\lambda_{Po}-\lambda_{Bi}}A_{Bi}(0)\left( \frac{{1-e}^{-(\lambda_{Bi}+\lambda_{b})T}}{\lambda_{Bi}+\lambda_{b}} - \frac{1-e^{-(\lambda_{Bi}+\lambda_{u})T}}{\lambda_{Bi}+\lambda_{u}}-\frac{1-e^{-(\lambda_{Po}+\lambda_{b})T}}{\lambda_{Po}+\lambda_{b}}+\frac{{1-e}^{-(\lambda_{Po}+\lambda_{u})T}}{\lambda_{Po}+\lambda_{u}} \right)$

⇒ $Ã_{Po}=\frac{\lambda_{Po}\lambda_{u}}{\lambda_{Po}-\lambda_{Bi}}BRA_{Bi}(0)\left( \frac{1}{(\lambda_{Bi}+\lambda_{b})(\lambda_{Bi}+\lambda_{u})}-\frac{1}{(\lambda_{Po}+\lambda_{b})(\lambda_{Po}+\lambda_{u})} \right)$

^209^Tl kinetics:

$A_{Tl}\left( t \right)=\frac{\lambda_{Tl}\lambda_{u}(1-BR)}{{(\lambda}_{u}-\lambda_{b})(\lambda_{Tl}-\lambda_{Bi})}A_{Bi}\left( 0 \right)\left( e^{-(\lambda_{Bi}+\lambda_{b})t}{-e}^{-(\lambda_{Bi}+\lambda_{u})t}-e^{-(\lambda_{Tl}+\lambda_{b})t}+e^{-(\lambda_{Tl}+\lambda_{u})t} \right)$⇒

$$Ã_{Tl}(T)=\frac{\lambda_{Tl}\lambda_{u}(1-BR)}{\lambda_{Tl}-\lambda_{Bi}}A_{Bi}(0)\left( \frac{{1-e}^{-(\lambda_{Bi}+\lambda_{b})T}}{\lambda_{Bi}+\lambda_{b}} - \frac{1-e^{-(\lambda_{Bi}+\lambda_{u})T}}{\lambda_{Bi}+\lambda_{u}}-\frac{1-e^{-(\lambda_{Tl}+\lambda_{b})t}}{\lambda_{Tl}+\lambda_{b}}+\frac{{1-e}^{-(\lambda_{Tl}+\lambda_{u})t}}{\lambda_{Tl}+\lambda_{u}} \right)$$

⇒ $Ã_{Tl}=\frac{\lambda_{Tl}\lambda_{u}(1-BR)}{\lambda_{Tl}-\lambda_{Bi}}A_{Bi}(0)\left( \frac{1}{(\lambda_{Bi}+\lambda_{b})(\lambda_{Bi}+\lambda_{u})}-\frac{1}{(\lambda_{Tl}+\lambda_{b})(\lambda_{Tl}+\lambda_{u})} \right)$

^209^Pb kinetics:

$A_{Pb}\left( t \right)=\lambda_{Pb}A_{Bi}\left( 0 \right) \frac{\lambda_{u}}{\lambda_{u}+\lambda_{b}}\times\left\{ \frac{\lambda_{Po}BR}{\left( \lambda_{Po}-\lambda_{Bi} \right)}\left( \frac{e^{-\left( \lambda_{Pb}+\lambda_{b} \right)t}-e^{-\left( \lambda_{Pb}+\lambda_{u} \right)t}-e^{-\left( \lambda_{Bi}+\lambda_{b} \right)t}+e^{-\left( \lambda_{Bi}+\lambda_{u} \right)t}}{\lambda_{Bi}-\lambda_{Pb}}+\frac{e^{-\left( \lambda_{Po}+\lambda_{b} \right)t}-e^{-\left( \lambda_{Po}+\lambda_{u} \right)t}-e^{-\left( \lambda_{Pb}+\lambda_{b} \right)t}+e^{-\left( \lambda_{Pb}+\lambda_{u} \right)t}}{\lambda_{Po}-\lambda_{Pb}} \right)+\frac{\lambda_{Tl}(1-BR)}{\left( \lambda_{Tl}-\lambda_{Bi} \right)}\left( \frac{e^{-\left( \lambda_{Pb}+\lambda_{b} \right)t}-e^{-\left( \lambda_{Pb}+\lambda_{u} \right)t}-e^{-\left( \lambda_{Bi}+\lambda_{b} \right)t}+e^{-\left( \lambda_{Bi}+\lambda_{u} \right)t}}{\lambda_{Bi}-\lambda_{Pb}}+\frac{e^{-\left( \lambda_{Tl}+\lambda_{b} \right)t}-e^{-\left( \lambda_{Tl}+\lambda_{u} \right)t}-e^{-\left( \lambda_{Pb}+\lambda_{b} \right)t}+e^{-\left( \lambda_{Pb}+\lambda_{u} \right)t}}{\lambda_{Tl}-\lambda_{Pb}} \right) \right\}$

⇒ $Ã_{Pb}= \lambda_{Pb}A_{Bi}\left( 0 \right)\lambda_{u}\times\left\{ \begin{aligned} \frac{\lambda_{Po}{BR}_{Po}}{\left( \lambda_{Po}-\lambda_{Bi} \right)}\left( \begin{aligned} \frac{1}{\left( \lambda_{Bi}-\lambda_{Pb} \right)\left( \lambda_{Pb}+\lambda_{b} \right)\left( \lambda_{Pb}+\lambda_{u} \right)} - \frac{1}{\left( \lambda_{Bi}-\lambda_{Pb} \right)\left( \lambda_{Bi}+\lambda_{b} \right)\left( \lambda_{Bi}+\lambda_{u} \right)} \\ + \frac{1}{\left( \lambda_{Po}-\lambda_{Pb} \right)\left( \lambda_{Po}+\lambda_{b} \right)\left( \lambda_{Po}+\lambda_{u} \right)} - \frac{1}{\left( \lambda_{Po}-\lambda_{Pb} \right)\left( \lambda_{Pb}+\lambda_{b} \right)\left( \lambda_{Pb}+\lambda_{u} \right)} \end{aligned} \right) \\ +\frac{\lambda_{Tl}{BR}_{Tl}}{\left( \lambda_{Tl}-\lambda_{Bi} \right)}\left( \begin{aligned} \frac{1}{\left( \lambda_{Bi}-\lambda_{Pb} \right)\left( \lambda_{Pb}+\lambda_{b} \right)\left( \lambda_{Pb}+\lambda_{u} \right)} - \frac{1}{\left( \lambda_{Bi}-\lambda_{Pb} \right)\left( \lambda_{Bi}+\lambda_{b} \right)\left( \lambda_{Bi}+\lambda_{u} \right)} \\ + \frac{1}{\left( \lambda_{Tl}-\lambda_{Pb} \right)\left( \lambda_{Tl}+\lambda_{b} \right)\left( \lambda_{Tl}+\lambda_{u} \right)} - \frac{1}{\left( \lambda_{Tl}-\lambda_{Pb} \right)\left( \lambda_{Pb}+\lambda_{b} \right)\left( \lambda_{Pb}+\lambda_{u} \right)} \end{aligned} \right) \end{aligned} \right\}$
